# Stories to Be Told: Korean Doctors Between Hwa-byung (Fire-Illness) and Depression, 1970–2011

**DOI:** 10.1007/s11013-012-9291-x

**Published:** 2012-12-11

**Authors:** Soyoung Suh

**Affiliations:** Department of History and Asian and Middle Eastern Studies Program, Dartmouth College, 404 Carson Hall, Hanover, NH 03755 USA

**Keywords:** Hwa-byung (Fire-illness), *han*, Psychiatry in Korea, Constrained-fire, Depression, Illness narratives

## Abstract

This article analyzes the process of the making of *hwa*-*byung* (fire illness) an internationally recognized term for a Korean emotion-related disorder. To index *hwa*-*byung* as a valid condition within professional medical circles, Koreans draw on both the traditional idea of “constrained fire” and the DSM’s modern identification of “depressive disorders.” Examining the research on *hwa*-*byung* since the 1970s, conducted by both Korean psychiatrists and doctors of traditional medicine, this article demonstrates how inextricably conceptions of Korean-ness in medicine have been tied to the right positioning of Korea in a global context. The project of defining a uniquely Korean malady reflects the desire of medical professionals to make the indigenous meaningful, thereby guaranteeing a tool for gaining circulation and foreign recognition. Studies of *hwa*-*byung* since the 2000s have in many ways been a reflection of the endeavor to interpret patients’ narratives as a therapeutic resource. Some *hwa*-*byung* specialists have dealt with patients’ stories of illness over time and argue for establishing better techniques of clinical communication. Whereas the label of *hwa*-*byung* initiated the indigenous turn during the 1980s, now the term succinctly responds to the recent trend of exploring the colloquial dimension of medicine. This also demonstrates the way in which *hwa*-*byung* has been (dis)assembled at the junction of global and domestic flows.

## Prologue

In the 1970s, Korean psychiatrists became puzzled by a malady commonly referred to as *hwa*-*byung* (fire-illness) (Min 2009a, pp.1–2). They reported that most patients who sought treatment were poor, middle-aged women with little formal education. The patients had often been tormented by a demoralized husband or a vicious mother-in-law for several decades and exhibited a range of psychosomatic symptoms such as anxiety, obsessive-compulsiveness, anorexia, and irritability. At first glance, Korean psychiatrists thought the symptoms similar to those of “neurotic disorders” (Min et al. 1986, p. 654), but the American Psychiatric Association’s standardized diagnostic manual, DSM-III, did not appear to have any information on hwa-byung. Korean psychiatrists then turned to their indigenous medical heritage in vain, finding that the major classics of Korean traditional medicine, such as Hŏ Chun’s (1546–1615) *Precious Mirror of Eastern Medicine* (Tongŭi pogam 東醫寶鑑, 1613) and Yi Che-ma’s (1838–1900) *Longevity and Life Preservation in Oriental Medicine* (Tongŭi susebowŏn 東醫壽世保元, 1894), did not recognize hwa-byung as a disease category.

The character “hwa” means fire, and hence alludes to centuries-old medical discussions about “fire” in East Asia (Li 2002). In particular, Chinese physicians from the twelfth century onwards began to emphasize the role of fire as an internal pathogenic agent, thereby articulating the medical understanding of human desires and emotions (Furth 2006). Sharing a textual tradition with China, Korea also adopted the Chinese articulation of fire from the sixteenth century on. Despite this long history of discourse on fire, hwa-byung was rarely mentioned as a disease category in Korean medical texts (Min 2009a, pp. 18–19).

The obscurity of hwa-byung as a textual label contrasts sharply with Koreans’ self-evident experience of the illness in their daily lives. Kim Yŏl-gyu, a scholar of Korean culture, depicts hwa-byung as Korea’s national illness (Kim 2004), while newspaper articles regularly use the term hwa-byung to contextualize the lamentable situation of women in contemporary Korea. For instance, *Chosŏn ilbo* reported that more than 80 % of women fighting various types of cancer are also diagnosed with hwa-byung (March 23, 2011). The condition not only affects anonymous women on the economic and cultural margins of Korean society, but also elite women like Kim Youngju, the wife of famed resistance fighter and national poet Kim Chi-ha. Kim Young-ju confessed that she had suffered from hwa-byung for decades before being cured by a practitioner of traditional medicine. She states, “I was sick every day. I felt like floating without any sensation in my lower body. After taking medicine prescribed by this elderly gentleman, I felt like a clod of constrained fire (*ulhwa* in Korean or *yuhuo* in Chinese, 鬱火)^1^ was suddenly cast out of my chest” (*Chosŏn ilbo*, Feb 28, 2011).

Besides contemporary women, the affliction has also been historically associated with men. One of the most well-known, reform-minded kings in Korean history, King Chŏngjo (1752–1800), is known to have perished due to an ulcer aggravated by years of hwa-byung. His constant complaints about the illness are revealed in court records of the time. Ten days before his death, the king stated:

This symptom is caused by hwa-byung accumulating for many years. I feel worse these days, but wasn’t able to resolve this problem. I do not want to talk about anything, even important issues. I also gradually feel sick and tired of meeting you vassals. The people in this court do not fear anything, so how can *fire*-*qi* not be clumping in my chest?” (*Veritable Records of the Chosŏn Dynasty*, June 16, 1800)

Hwa-byung first appeared in Korean court records in 1603, and a series of terms implying fire-related discomforts showed up more than 50 times between the seventeenth and late nineteenth centuries.^2^ Although medical texts did not explicitly recognize hwa-byung as a name of a disease, past court records and the contemporary circulation of the term provide justification for its reputation as Korea’s “national illness.”

Hwa-byung’s ostensible ubiquity in Korean society, in contrast to its relative scarcity in professional medical discourse, has provoked various efforts to officially label the problem. In addition to Korean psychiatrists, doctors of traditional medicine have also actively joined the effort over the last 30 years to define, diagnose, and cure the affliction Koreans know as hwa-byung. The advance of depression as a global disease (Watters 2010)—combined with Korea’s rising suicide rate (*Han’gyŏre sinmun*, Sep 19, 2006)—has encouraged Korean medical professionals to study cultural factors that may be detrimental to the mental health of people in Korea. This article aims to analyze the process through which hwa-byung draws on both the traditional idea of “constrained fire” and the DSM’s modern identification of “depressive disorders” to establish hwa-byung as a valid condition within professional medical circles.

Medical research on hwa-byung has increased rapidly, borrowing terms and theories from across conventional disciplinary boundaries, including traditional medicine, psychiatry, psychology, and medical statistics. The process of labeling an indigenous illness for a broader audience reflects the desire of Korean professionals to take the initiative in their field at an international level, and to be seen as both modern and advanced. Simultaneously, various interpretations of hwa-byung have emerged and then disappeared without fully stabilizing its definition in medical writings. This article aims to depict the way hwa-byung has been (dis)assembled at the junction of global and domestic flows. How does an experience that is believed to be indigenous gain a classification applicable to both domestic and foreign cases? What sources did medical professionals utilize to index Koreans’ particular experience in the supposedly universal grid of knowledge? Given the inventive nature of hwa-byung, how can we understand the incessant circulation of the label in contemporary Korea? Finally, but not least important, what does this process tell us about the Korean pattern of producing medical knowledge about local experience at the margins of a Western-centered psychiatry?

Past research in Anglophone academia has rarely put hwa-byung in its historical context. Most medical studies have defined it in accordance with the scheme of contemporary psychiatric etiology; hence, hwa-byung has been compared to other psychiatric syndromes for identification purposes.^3^ With a growing interest in multiculturalism, manifestations of hwa-byung among Korean-Americans have been studied as part of the Asian-American psychiatric experience (Pang 1990; Lin et al. 1992). Past studies, however, have not fully analyzed the way hwa-byung has been continually transformed by new research findings, nor have they considered the clinical implications arising from the challenges of interdisciplinary study and international recognition.

This article does not aim to provide another report on hwa-byung’s definition, manifestation, and treatment. Rather, this study examines hwa-byung as part of the changing mode of ethnic/indigenous portraits that Korean medical professionals use to solidify their knowledge and status and to strengthen their clinical strategies. Essentializing the nature of the autochthonous experience in medicine does not reflect the major concern of this research. As Kapil Raj’s study concerning botanical exchange between India and Europe reveals, purely indigenous forms of knowledge that fail to register with central medical conventions cannot be communicated outside the locale (Raj 2006). The medical and scientific studies about the indigenous are shaped and modified, reflecting the interest of the intellectual and cultural center. Accordingly, the story of hwa-byung demonstrates that medical writing at the margins reveals the desire to catch up with the latest agendas and conventions of the center. Terms and idioms produced at the center are carefully selected to appeal to audiences in both the cosmopolitan center and the local context. Therefore, narrating indigenous experience often results in shifting strategies, leading to questions such as to what extent should the peculiarities of the indigenous be emphasized or tailored. Sensitive to their positions from a global standard, Korean medical professionals have been both ambitious and ambiguous in documenting local experience for more authoritative audiences.

This article also intends to scrutinize the way in which a few doctors utilize the label of hwa-byung as a tool for criticizing the current state of Korean medicine, which they view as reductionist and corporatized. Publishing monographs of advice on hwa-byung, recent specialists have begun to argue for a better relationship between doctors and patients. Including many first-hand stories about their patients, Korean hwa-byung specialists come to highlight life complexity, individuality of illness, and the significance of doctors’ compassion with their patients. Interestingly, these popular texts about hwa-byung remain eclectic in defining the illness and suggesting treatments. For instance, some traditional doctors equate hwa-byung with stress, whereas others equate it with depression. The treatments also vary according to doctors’ educational backgrounds and institutional affiliations. However, all the popular advice shares a common element in highlighting narratives as a crucial part of the curing process. To treat hwa-byung fully, a Korean doctor had to first be a listener and then had to represent their patients’ illness stories, which can hardly be reduced to tables, numbers, and simple questionnaires. By listening to patients’ stories over time, doctors also accumulated their own narratives about clinical intervention, which cannot be succinctly summed up in the form of a scientific article or medical report. Resonating with what Arthur Kleinman calls “mini-ethnography” (Kleinman 1988) and Rita Charon’s idea of “narrative medicine” (Charon 2006), the contemporary Korean hwa-byung discourse serves as a means to expose criticism about existing clinical problems and to conjure up a vision for better clinical communication.

Considering the evolution of how Korean medical professionals have narrated indigenous experience from the 1970s onwards, this article examines the enduring yet unfulfilled desires among doctors, which have also shaped the diagnostic approaches to Koreans’ emotional discomfort. The medical representation of hwa-byung has been flexible, overlapping, and sometimes self-effacing, alluding to those professionals’ manifold strategies at the global and domestic junctures.

### From Neurosis to Anger Disorder

Yi Shi-Hyung was the first to posit Korean culture as a key factor in determining the etiology of hwa-byung.^4^ Several previous authors had discussed the different manner in which lay people in the countryside and professionals in psychiatric clinics conceptualized hwa-byung. Yi went one step further by highlighting unfavorable socio-cultural conditions in Korea at large. Based on his clinical encounters with female patients, Yi specifically depicted hwa-byung as the sickness of an oppressed society in which marginalized women find few means to express their desires and resentment (Yi 1977). With the notion of a distinct cultural illness now in place, Min Sung-kil, a psychiatrist affiliated with Yonsei University, one of the most privileged private universities in Korea, next began to search for a means to translate what Korean patients and their families reported to be hwa-byung into the language of psychiatry in order to establish an objective diagnostic standard.^5^ Min began by interviewing 287 outpatients who visited psychiatric clinics in three different locations in Korea for treatment of what was then diagnosed as “neurosis” (Min et al. 1986, p. 653). To conduct these interviews, he relied on the “Diagnostic Interview Schedule, 3rd edition,” a tool designed by the National Mental Health Institute (NIMH) of the United States but subsequently translated into Korean and validated for use with Korean patients. Min relied on a standardized interview schedule to maximize the reliability of his data. Yet, he acknowledged his own modification of it by deleting what he considered to be irrelevant questions while adding others.^6^ In an attempt to garner more international recognition for his modified schedule, he also invited an American psychiatrist from the Pacific/Asian-American Mental Health Research Center to appropriately train his Korean interviewers (Min et al. 1986, p. 655).

Out of 287 patients, 56 were classified as hwa-byung, with both the patients and their families agreeing with the diagnosis. In a similar fashion, 157 were identified as the non-hwa-byung group. According to the diagnostic categories of the DSM-III, the hwa-byung patients turned out to belong to multiple diagnostic categories, whereas the non-hwa-byung patients had one or no diagnosis. The most frequently found associated diagnosis for hwa-byung patients was depression (major depression and dysthymic disorder, alone or combined) and somatization disorders combined (Min et al. 1986, p. 661). The overlapping of diagnoses was the major characteristic of hwa-byung.

To support his finding, Min carried out another empirical research study on the isolated Bokil Island. Remotely placed near the southwestern coast of Korea, residents on the island work mostly in agriculture and are assumed by Min to preserve their indigenous culture more successfully than urban residents (Min 1986). Among 562 surveyed outpatients, Min’s team was able to interview 138 individuals, identifying 34 as belonging to the hwa-byung group and 67 as belonging to the non-hwa-byung group. Five trained interviewers applied the DIS-III and DSM-III diagnostic standards. The diagnosis of hwa-byung primarily depended on patients’ answers to the questions the interviewers asked. The research outcome supported Min’s earlier research among outpatients in urban settings. Hwa-byung patients qualified for two or more combined diagnoses. Min argued that “hwa-byung is a syndrome with symptoms sufficiently severe to be diagnosed as neurotic disorders” and it includes “somatization, anxiety, and depression combined” (Min 1986, p. 466).

Min also acknowledged the weak point of his research. Patients’ subjective statements were the only grounds for identifying hwa-byung (Min 1991, p. 1194). Patients were often confused and inconsistent in remembering how serious their psychosomatic problems were and misunderstood terms the psychiatrists used in the interviews (Min 1986, p. 464). Some patients’ testimonies slipped out of the diagnostic classification of DSM-III. More fundamentally, he found that the cultural-linguistic barrier hampered the universal application of psychiatric terminologies. Min states:

It is problematic that our country’s psychiatrists rely solely on theories of Western medicine in treating our country’s patients. Socio-cultural factors that cause our patients’ mental illnesses are different from those of (Western) societies. (Our patients’) manifestation of symptoms, ways of expressing them, and methods of treatments cannot be separated from the traditional attributes of our family, society and culture. More to the point, genetic and constitutional difference may not be fully conceptualized by a Western standard. Our country is developing into a Western industrial society, and the term hwa-byung is becoming less familiar to most Koreans. However, the socio-cultural attributes of our traditional society will continue to have their repercussions beneath the rapidly changing environment on the surface (Min 1986, p. 653).

Min did not propose overcoming the limitations of his research by improving his interviewing skills. Instead, he expressed his dissatisfaction with the standardized psychiatric disease categories, which are American in origin. Because he believed that modern psychiatry is too entangled in its own cultural origins, he found the identification of hwa-byung elusive. Min continued to express his criticism of the limits of psychiatric language throughout the early 1990s.

Given this predicament, Min longed to find what he considered “genuinely” Korean factors that may affect mental health. He felt that Korean psychiatrists could not consider the development of symptoms, expressiveness, and the treatment without digging thoroughly into Korean family structure and socio-cultural factors. Inasmuch as psychiatrists need to know an individual patient’s deep-rooted life stories for treatment, the so-called “uniquely” Korean way of life should be examined by (Korean) psychiatrists in order to design the appropriate treatment. This partially explains why Min became interested in *han* (恨), the supposedly Korean way of holding grudges and experiencing anger. According to Min, hwa-byung and *han* have an etiological relationship. Interviewing 146 outpatients who visited the clinic of psychiatrics at Yonsei Severance Hospital to treat their hwa-byung, he was able to question 125 about their *han*, and 117 answered that *han*, to some degree, is relevant to hwa-byung (Min 1991, p. 1192).

Unpleasant experiences in life, Min argued, cause either hwa-byung or *han*. The difference mostly lies in time duration and the inclusion of socio-political vicissitudes. Whereas hwa-byung addresses comparatively recent losses and the despair of an individual, the causes of *han* stem from decades ago, such as personal difficulties due to Japanese colonization (1910–1945) or the Korean War (1950–1953). The most frequently reported emotional reactions among hwa-byung with *han* patients are “mortification,” “frustration,” “nihilistic mood,” “anxiety,” and “regret.” (Min 1991, p. 1189) Although *han* helped to explain the origins of hwa-byung, Min realized it caused other problems with his research framework. For instance, those who have *han* without any hwa-byung would need to be further examined to elaborate on this etiological relationship. The impact of stress on *han* might provide another meaningful point of reference for understanding the relationship between *han* and hwa-byung. The comparison between *han* and *chŏng*, another indigenous term for the Korean feeling of emotion, should also be examined. Despite the need for further research, Min never hesitated to conclude that “hwa-byung can be said to be a pathological condition of *han* resulting from the failure to overcome it in the long term” (Min 1991, p. 1198). Min felt that *han* could provide an interpretative frame for understanding Koreans’ collective responses to the accumulated plight.

Needless to say, Min scarcely questioned whether *han* could speak for the nation. He viewed *han* as a uniquely national (Han’guk minjok, 韓國民族) expression of suffering (Min 1991, p. 1195). To properly treat this problem, he called for an ethnic psychiatry that deals with the Korean “nation’s neuro-psychiatry” (minjok chŏngsin ŭihak 民族精神醫學). Min’s elaboration of *han* as an etiology for hwa-byung continues to be quoted by both psychiatrists and doctors of traditional medicine.^7^ Today, Min’s work is recognized as playing a significant role in establishing *han* as an icon of the uniquely Korean psychological state.

The growing number of studies of *han* produced by Korean historians, theologians, and literary critics up until the 1990s have provided a substantial repertoire for Min to argue the etiological significance of *han*. *Han* represents the quintessential element of Korean aesthetics. From the 1970s, literary critics began to describe *han* as the most prevalent theme of Korean literary expressions. From the oldest poem to the most contemporary novel, *han* best exhibits Korean’s deep-rooted emotional foundation (Ch’ŏn 1993). *Minjung* theology, an indigenous Christian theology that came out of the Korean human rights movement and social activism during the 1960s, also interpreted *han* as that which evolved from the Korean experience of the oppressed (Lee 1994; Son 1984; Kim 1994; Park et al. 1997). Studies on Korean music trace the origin and modification of sound that expresses *han* (Willoughby 2002). Inquiries into Korean shamanism never fail to point out that *han* became the indispensable cause and result of being drawn to shamanism (Ch’oe 1991, 1994; Kim 1999).

It is interesting to see that different avenues of Korean discourse on *han* share a twofold desire to situate *han* in a specific time and place while simultaneously universalizing it. *Han* needs to be articulated through Korea’s own history. For instance, Ko Ŭn, a well-known poet and thinker whose ideas represent major claims of the nationalist movement in Korean literature, put the origin of *han* in ancient Korea, in Kochosŏn (古朝鮮, circ.1000–108 BCE), and surveyed *han*’s enduring manifestation through the socio-political vicissitudes in the period of the Three Kingdoms (circa 57 BCE–668 ACE), the Koryŏ (高麗, 918–1392 ACE), and Chosŏn dynasties (朝鮮, 1392–1910 ACE). In parallel with *han*’s historicity, however, Ko also explores *han*’s general validity. *Han* is compared with the Chinese idea of “*hen*” (恨), the Mongolian feeling of “Horosul,” the Manchurian expression of “Krosocuka,” and the Japanese meaning of “Oorami.” This comparative approach, in a sense, puts *han* in a broader context by encompassing other peoples’ experience of suffering. *Han* needs to go beyond Korea’s geo-political boundaries to resonate with other expressions of the oppressed. In other words, these discussions on *han* from the non-psychiatric disciplines expose the fact that the Korean elaboration of “Koreanness” is paralleled by the desire to go beyond the autochthonous. The idea of the indigenous requires a broader register for comparison and circulation.

In parallel, the studies on the indigenous conditions for mental health were not entirely oriented toward domestic demands. Uniquely Korean narratives are inclined toward audiences in the outer world. In retrospect, Min confessed that hwa-byung was welcomed more by international audiences than by domestic listeners. Psychiatrists abroad found a Korean case of culture-bound syndrome meaningful (Min 2009a, p. 219). In addition, Min acknowledged that American psychiatrists’ research prior to his own studies actually motivated him to take the initiative to report hwa-byung to global readers. Min viewed Keh-ming Lin’s article published in the *American Journal of Psychiatry* in 1983 as the first international report on hwa-byung (Lin 1983). “How come an American psychiatrist is reporting about Korean hwa-byung?” he wondered (Min 2009a, p. 217). Feeling challenged by Lin, Min began to collect clinical cases about hwa-byung.

Min’s reputation has flourished along with his theories related to hwa-byung. From the late 1990s onward, Min has presented his work at numerous conferences held by international societies, such as the “Association of Korean-American Psychiatrists,” the “American Psychiatric Association,” the “World Psychiatric Association,” the “Pacific Rim College of Psychiatrists,” the “World Association of Social Psychiatry,” and the “World Congress of Cultural Psychiatry” (Min 2009a, pp. 220–222).

The fourth edition of the DSM (DSM-IV) recognized hwa-byung as a culture-bound syndrome in 1994. Following this success, the goals of Korean psychiatric research have become focused on gaining even more international recognition. Biological research, anger-related brain research, development of hwa-byung scale, and drug tests have aimed to verify hwa-byung’s autonomous existence (Min 2009b, pp. 220–222). Min has put more emphasis on characterizing hwa-byung as an anger disorder, which may be applicable to patients across cultural-ethnic boundaries (Min 2009a, p. 176). Min’s statistically sophisticated research in 2009 argued that hwa-byung could be meaningfully distinguished from symptoms of major depression and should therefore be elaborated on under another novel name, such as an anger disorder (Min 2009b, p. 77). He states, “Hwa-byung, according to biological, psychological, and social model, can be viewed as a psychiatric barrier that has both emotional and somatic/behavior symptoms relevant to anger. Hwa-byung can gain a name and be conceptualized in a DSM clinical category” (Min 2009b, p. 83).

One paradox with these efforts to universalize hwa-byung as an anger disorder is that they undermine the uniquely Korean cultural etiology of *han*. Min has called for further investigation of attributes associated with anger that are found in many cultures, such as “ataques de nervios” among Hispanics (Min 2009a, pp. 180–181). Ironically, Min’s earlier passion to make the Koreanness of hwa-byung visible has now led to his willing extinction of that distinction. Beginning with a passion to articulate the culture-bound attributes of hwa-byung in the early 1980s, Min ended up with a culture-neutral conceptualization of hwa-byung in the twenty-first century.

In his conceptualization of hwa-byung, Min favorably referred to perspectives of traditional medicine. Min’s earlier survey reveals that doctors of traditional medicine were mostly reluctant to accept hwa-byung’s close relationship with factors of Korean culture, such as *han*, while unanimously believing the autonomous status of the illness as a clinical entity. Korean psychiatrists, in contrast, paid more attention to indigenous culture as an etiological factor, yet were reluctant to define hwa-byung as a clinical category (Min et al. 1989, pp. 146–154). How did hwa-byung gain its currency among doctors of traditional medicine? The discussion below turns to doctors of traditional medicine who adopted changing strategies to help establish hwa-byung as a meaningful diagnostic category.

### From *Yu* to Depression

From the early 1990s onward, hwa-byung emerged as a significant research topic of traditional medicine.^8^ Psychiatrists’ endeavors to examine indigenous etiological factors seemingly challenged the professionals of traditional medicine (Kim et al. 2007, pp. 6–7). More to the point, traditional medicine itself became more specialized during this period, and thus, rushed to establish disciplines of subspecialty, such as pediatric, gynecological, and psychiatric associations.^9^ The *Journal of Oriental Neuropsychiatry*, for instance, was first published in 1990. The ascendancy of depressive disorders on a global scale has also motivated traditional medicine to further delve into hwa-byung. According to a government survey, the prescription of antidepressant medication in South Korea increased by 52.3 % between 2004 and 2008. Nearly a fifth of women in South Korea are reported to suffer from depression at some point in their lives, and female use of antidepressant medication was double the male use between 2004 and 2008 (*Yŏnhamnyusŭ*, July 26, 2009). Accordingly, the Korean Society for Depressive and Bipolar Disorders was first established in 2001.^10^ Traditional medicine’s growing interest in hwa-byung should be understood in this milieu of pressing needs to resolve emerging mental health issues in both domestic and global settings. The number of articles containing the word “depression” or “hwa-byung” in the *Journal of Oriental Neuropsychiatry* increased substantially after 2000. Following the initiative of Korean psychiatrists, the specialization of traditional medicine, and the advance of depression, more and more clinics marketed their expertise in treating hwa-byung.

Depression and hwa-byung in their Korean nomenclatures share a common character, *yu* (鬱), which has long been a significant etiological concept in traditional medicine in East Asia. Translated into “constraint,” the term *yu* primarily implies something that is stuck, or unable to move or change, because of a blocked *qi* movement. In East Asian medicine, the pathological condition of *yu* can frequently be a cause of fire, and this relationship between *yu* and fire is captured in another common name for fire illness, ulhwa-byung (*yuhuobing* in Chinese 鬱火病). As Volker Scheid demonstrates well in this volume, “*yu*” has evolved over time and has been associated with various illness conditions. Thus, *yu* cannot be succinctly reduced to any single illness or disease category. The rich textual repertoire and multiple clinical implications of “*yu*” actually empowered Korean doctors of traditional medicine to enter the discourse surrounding hwa-byung. As latecomers to hwa-byung studies, the professionals of traditional medicine sought primarily to establish their unique diagnostic and clinical approaches by fleshing out the traditional ideas about “*yu*” and fire (*hwa/huo*) (J. Kim 2007, pp. 109–110).

In his earlier article, Kim Jong-woo, one of the most well-known hwa-byung specialists, argued that traditional medicine provides a better explanation of the four main questions about hwa-byung: (1) why hwa-byung is caused by upset, oppression, resentment, anger, and loathing; (2) why women are more vulnerable to hwa-byung; (3) why symptoms of hwa-byung seem similar to the traditional discourse on fire; and (4) why the pathogenic process involves a chronic disease.^11^ While elaborating on those four issues, Kim put forth the liver as central to the pathology of hwa-byung. Whereas Korean psychiatrists take the heart as the symbolic locus of hwa-byung, thereby linking it to a neuropsychiatric problem, Kim differentiated his perspective with the liver (Kim and Whang 1994, pp. 9–10). Kim explained that once the liver loses its distinctive function to regulate the activities of *qi*, as the result of excessive anger and frustration, the constraint of liver-*qi* (肝氣鬱結) tends to occur. If the liver-*qi* is blocked, then the *qi* probably transforms into heat-fire (熱火), thereby leading to an upward counter flow (肝火上逆) that resembles the symptoms of hwa-byung. Kim argued that this explains women’s fragility to hwa-byung. Due to women’s reproductive function, the liver, spleen, kidney, and two *jingmai*, *chongmai* (衝脈) and *renmai* (任脈), become particularly important for a woman’s health. When women reach menopause, both *jingmai* become weak, the *qi* of the kidneys decreases, and the internal *qi* of fire becomes easily agitated. This is called the “emptiness of kidney and agitation of fire (腎虛火動)” (J. Kim and Whang 1994, pp. 11–13).

Kim applied other pathologies, such as “rising heart fire (心火上炎),” “excessive liver fire (肝火亢盛),” and “flaming stomach fire (胃火熾盛),” to explain fire as an agent that determines other disease states (Kim and Whang 1994, p. 11). He argued that fire is the *qi* of upward tendency (上升之氣), always drying up yin-*qi*, thereby creating the wind (風), moving the blood (血), and causing tumors. Furthermore, fire interacts with the heart, and therefore the evil factor of fire disturbs the integrity of the mind-spirit (心神). Kim viewed hwa-byung as immoderate emotions that turned into excessive fire *qi* (五志過極化火). Based on these terms and reasoning, Kim argues that traditional medicine provides better pathological and etiological explanations for hwa-byung. To Kim, hwa-byung is neither a mere syndrome nor a psychosomatic disorder. It is an illness with its own unique pathological process, and hence, cannot be equated to stress or hysteria.

Kim’s composition of hwa-byung draws on a couple of referential sources. First, his wording of yin-yang and Five Phases directly refers to the Chinese classic, *Yellow Emperor’s Inner Canon* (*Huangdi Neijing*, *Inner Canon* hereafter), and its correlative construction of the human body. Kim also relies on contemporary textbooks of Korean traditional medicine, which put emphasis on *Inner Canon*’s holistic worldview. Kim’s understanding of fire is definitely drawn from the fourteenth century’s Chinese physician, Zhu Zhenheng’s (朱震亨, 1281–1358) conceptualization of fire as an agent of inner physiology. Zhu’s idea of nourishing yin to balance excess yang was highlighted by Kim through *Precious Mirror of Eastern Medicine* (Tongŭi pogam東醫寶鑑, 1613), which is considered to be one of the first distinctively Korean medical texts. *Precious Mirror* continues to be the major reference for contemporary Korean doctors of traditional medicine.

The last important reference for Kim is today’s traditional Chinese medicine (TCM) textbooks from China, which are now available in Korea. Through his investigation of these textbooks, Kim discovered *Mr. Zhang’s* *Essentials of Medicine* (張氏醫通), written by Zhang Lu (張璐), who lived in seventeenth-century Jiangsu China. Previously, Zhang had not been influential in Korea, yet mediated by TCM textbooks, Kim was able to draw on ideas from *Mr. Zhang’s* *Essentials of Medicine* to develop hwa-byung. For example, his interest in the manifestations of women’s *yin qi* related diseases came from these textbook presentations of Zhang. Kim also relied on TCM textbooks from modern China for information about fire’s physiological and pathological functions. Perhaps most importantly, Kim utilized the TCM textbook methodology of “pattern recognition and treatment determination, or *bianzheng lunzhi* 辨證論治,” as we will see below. It was not uncommon for Korean doctors of traditional medicine during the 1990s to refer to textbooks, journal articles, and monographs from Taiwan and China.

The textual resources for a Korean construction of hwa-byung, thus, include Korean psychiatrists’ early research on hwa-byung, quotes from Chinese classics like *Inner Canon*, a well-known Korean text like *Precious Mirror* (which summed up a fourteenth-century Chinese elaboration on fire), and contemporary TCM textbook views of fire as a physiological process. Korean medicine doctors, as latecomers to the treatment of hwa-byung in Korea, benefitted from TCM’s resources, thereby enhancing the rationale of using the “traditional” in treating hwa-byung.

Kim’s eclectic composition, however, did not last for long. Relying on the language of modern TCM from China, his research seemed to lack a specific Korean ethnic distinction. It became obvious that Kim’s strategic composition of hwa-byung has a limit in gaining an audience outside Korea. Unlike Min, who made the “Korean” illness visible to international audiences and actively circulated his work outside Korea, Kim worked with hardly any non-Korean psychiatrists, or with any Chinese or Japanese doctors of traditional medicine. Kim recognized the weakness of articulating hwa-byung solely based on the textual language of traditional medicine. Hwa-byung necessitated a different kind of linguistic composition.

To overcome these limitations, Kim turned to “objective” measurements, which he thought would eventually demonstrate traditional medicine’s successful diagnosis and treatment of hwa-byung and thereby gain a broader audience. Kim underlines, “No matter how diligently you may explain hwa-byung, if it is not tested by biological and scientific methods, the research can hardly be widely credited (Kim 2007).” Kim evolved toward a more advanced technique to quantify hwa-byung. For instance, in the early 2000s, Kim took a position that viewed hwa-byung as a phenomenon related to stress, which enabled him to measure the development of symptoms using an officially acknowledged scale (Lim et al. 2000). Relying on and slightly modifying the GARS (Global Assessment of Recent Stress), Kim applied more advanced statistical methods. Forty-eight outpatients were surveyed, and their answers were analyzed by paired sample *t* test. Kim referred to T. H. Holmes’s “The Social Readjustment Rating Scale,” published in 1967, and Camile Lloyd’s “Life Events and Depressive Disorder Reviewed: Events as Predisposing Factors,” published in 1980.^12^ Kim emulated the scale designs of psychological and psychosomatic research.

During the 2000s, Kim shifted away from stress toward depressive disorders. By equating *yu* with depression, Kim found an effective way to objectify hwa-byung. Kim’s growing emphasis on a standardized diagnostic manual of traditional medicine suitably exhibits the overall trend to identify *yu* with depression in Korea. After years of interdisciplinary collaboration, Kim was able to develop a “hwa-byung diagnostic interview schedule (HBDIS),” which was supported by more sophisticated statistical methods. Verified with validity and credibility, his scale was approved by the Korean Psychological Association in 2004. Based on this scale, Kim was able to make his own argument with a more competent tone (Lee et al. 2005). Although earlier research in psychiatry described hwa-byung as a combination of multiple symptoms or a strong manifestation of somatic disorders, Kim underlines that more than 70 % of his hwa-byung sample patients turned out to be diagnosed according to DSM-IV’s major depression disorder and dysthymic disorder (Lee et al. 2005, p. 12).

Kim’s study of hwa-byung as a major depressive disorder also drew on TCM research models from China, in particular, using the clinical standardizations of “pattern recognition and treatment determination (*bianzheng lunzhi* 辨證論治, “pattern recognition” hereafter).” As Eric Karchmer has demonstrated, since the late 1950s, “pattern recognition” has been central to codifying the uniqueness of Chinese medicine while also providing a technique, often unacknowledged, for integrating Chinese medicine with biomedicine (Karchmer 2010, pp. 226–230). Korean practitioners have also recognized the significance of “pattern recognition,” and since 1995 they have been working on their own set of standardizations, called “Oriental Medicine Standard-Prime,” which is now marketed online.^13^

In his 2007 article, Kim compared 17 hwa-byung-only patients with 20 patients from the hwa-byung and major depression double diagnoses group. For this sample selection, Kim used standardized scales, such as HBDIS, SCID, OMS-prime, the Hamilton Rating Scale for Depression (HRDS), and the Montgomery-Asberg Depression Rating Scale (MADRS). Kim examined socio-demographic data and then analyzed two groups’ “pattern recognition.” He found no significant differences in the distribution of patients according to major patterns between the two groups. Examined by OMS-prime, both groups have a tendency to show “deficiency of yin and yang of the heart” most frequently. Based on this analysis, Kim concluded that “hwa-byung is a syndrome that has many different symptoms, but there is no difference between the hwa-byung group and hwa-byung with major depression group (double diagnosis) in terms of standardized pattern recognition.” Because Kim’s research aimed to demonstrate overlaps where others, such as Min and other Korean psychiatrists, see differences, Kim argued that his approach to the study of “hwa-byung could be a new model for researching depression in Korea.” Kim added that Koreans have a tendency to experience more somaticized symptoms of major depression and express the depression through the language of hwa-byung (Kim et al. 2007, p. 12).

Kim further developed his comparison between hwa-byung and depression. He suggested:

Although hwa-byung and depression are often considered as a single and separate disease entity (chilbyŏng 疾病), if traditional medicine’s method of pattern identification is applied, hwa-byung and depression can be conceptualized as a series of various symptoms respectively, but not separate illness entities. This is due to traditional medicine’s and Western medicine’s fundamentally different approaches to disease. And this indicates that Western medicine requires complementary research on symptomatic approaches, whereas traditional medicine necessitates further understanding about a disease-centered approach.” (Kim et al. 2007, p. 12)

Although Kim contrasted traditional medicine with its Western counterpart, he never posited traditional medicine as an alternative or complement to a psychiatric (scientific) approach. Rather, Kim strongly embraced the language of psychiatry, psychology, and statistics, thereby portraying his own version of traditional medicine that was well synchronized with the objective standardizations of biomedicine.^14^

The Korean identification of hwa-byung with depression has been further enhanced by a series of psycho-pharmaceutical research about traditional prescriptions. Published mostly after 200 0, those articles equated depression with *yu*-related illness, to which hwa-byung also belongs. By identifying depression with hwa-byung, those articles were able to apply the experimental model of antidepressants to Korean traditional prescriptions, thereby objectively elaborating hwa-byung as a medical entity. Chung, for instance, examined “Coptis Decoction to Resolve Toxicity (huang lian jie du tang 黃連解毒湯),” one of the well-known prescriptions for hwa-byung in South Korea. Chung states that “in order to treat depression, traditional medicine can rely on prescriptions that treat fire (pathologies)” (Chung and Kim 2003, p. 2). In addition to Chung’s finding, substantial research has been carried out since 2000 to emulate the psycho-pharmaceutical research about antidepressants.^15^

The previous analysis traces the way hwa-byung has been defined, diagnosed, and treated by both Korean psychiatrists and doctors of traditional medicine since the 1970s. To make hwa-byung sensible in medical discourse, professionals across fields have utilized various linguistic and numerical resources and have strategically modified hwa-byung’s relationship with depressive disorders. Needless to say, hwa-byung’s autonomous status as an illness entity is related to the authorization of medical professionals and their career building efforts.

Kim began his research to differentiate traditional medicine’s unique approach to hwa-byung in terms of both diagnosis and treatment (Kim and Whang 1994). As time went by, Kim relied more on quantitative methods and less on the language of traditional medicine, aiming to objectively demonstrate hwa-byung’s autonomous existence. The advance of depression as a global disease stimulated traditional medicine’s equation of *yu* with depressive disorders, thereby providing a link to interpret hwa-byung as the Korean version of depression. Although both Kim and Min agreed that more quantitative and interdisciplinary research was indispensable to fully understanding hwa-byung, Min and Kim diverged in predicting the future of hwa-byung in a global context. While Min argued for transforming hwa-byung into an anger disorder, thereby differentiating it from depression (Min 2011, 2012), Kim was drawn to equating the illness with depression to a certain degree.

Both Min and Kim successfully responded to the rise of cultural psychiatry, thereby making the “Korean” illness visible to international audiences. Although the idea of a uniquely Korean malady first encouraged Min and Kim’s research, both of them eventually distanced themselves from the ethnic grounding of hwa-byung. Although Min first celebrated the locally specific experience of the Korean psyche, thereby revealing the cultural bias of American psychiatry, he eventually came to argue for a universal label for hwa-byung that goes beyond Korea’s own cultural-geographical boundary. In a similar vein, Kim first delved into the millennium-old textual heritage of traditional medicine, drawing on the language of the *Inner Canon*, renowned Chinese physicians in the thirteenth century, centuries-old Korean medical scholarship, and modern TCM. However, Kim strongly desired to have hwa-byung recognized by contemporary psychologists, pharmacologists, and psychiatrists; thus, he had to remake hwa-byung into an entity that could circulate across cultural, national, and disciplinary boundaries.

What medical research about hwa-byung tells us is that there are no essentially Korean features of the mental disorder. Rather, medical professionals’ reports on Koreanness illustrate the process through which a biography of the local has emerged and been modified, disclosing its (dis)juncture with global trends. In spite of its unstable foundations of indigenity, hwa-byung continues to represent “Korean” emotional disorders. A series of recent publications about hwa-byung for popular audiences exposes the way in which experts’ academic research, epitomized by Min’s and Kim’s cases, is translated into non-academic terms. Tracing immediate and personal voices of hwa-byung specialists outside academia, the next section asks, “What does the continuing focus on the concept of hwa-byung mean for Korean doctors?” Simply put, why does “hwa-byung” continue to be popular in contemporary Korea, given the lack of any explicit consensus among doctors? What possibility does the label of hwa-byung open for doctors?

### Toward a Better Communicative Sphere

Since 1997, almost a dozen popular texts about hwa-byung have been published. Min and Kim’s research articles were edited then published in monographs in 2009 and 2007, respectively. In addition, since 2006, more than five new volumes about hwa-byung were introduced by doctors of traditional medicine. With their clinical experience and personal endeavor, these primers aptly expose hwa-byung specialists’ diagnostic strategies and methods of treatments. As non-academic publications, the current volumes do not detail their sources of references. However, their (dis)similar voices enable us to view the various ways in which the research initiatives of Min and Kim are emulated and modified by other medical experts, thereby allowing consideration of the function of hwa-byung as a textual label in expanding and limiting the scope of contemporary management of emotional disorders. Four authors and their texts will be examined in the below.

Hwang Ŭi-wan, as a well-established hwa-byung specialist, has treated patients for more than 40 years. He served as chair of the Department of Neuro-Psychiatry at the Hospital of Traditional Medicine at Kyung Hee University and as president of the Korean Society of Oriental Neuropsychiatry (*Daehan hanbang sin’gyŏng chŏngsin kwahakhoe*) (Hwang 2011). Hwang acknowledges that hwa-byung has never signified a single disease category in traditional medicine; however, the term has long been signified a disease name in Korea (Hwang 2011, p. 11). As Hwang co-authored a couple of articles with Kim, Hwang’s definition of hwa-byung and explanation of symptoms quite overlaps with Kim’s earlier findings.^16^

Ch’oe Yung-jin and his co-authors begin their monograph by defining hwa-byung as “uniquely Korean disease,” then ascribed the major cause to the socio-cultural environment of the country. Ch’oe argues that hwa-byung has accompanied Korea’s long history; thus, traditional medicine that has been a part of Koreans lives for millennia is better situated to deal with the illness. As an alumnus of Kyung Hee University, one of the top school of traditional medicine in Korea, Ch’oe shares a common institutional background with Hwang and Kim. Accordingly, the major reasoning and rhetoric Ch’oe develops for diagnosis and treatment is not radically different from that of Kim and Hwang.

Yun Yong-sŏp, the third author, majored in pharmaceutics at Ch’ungnam University in Korea, then graduated from Nanjing University of Medicine (南京中醫藥大學) in China in 1997. As the Korean government acknowledges only the graduates of domestic institutions of traditional medicine for licensure, Yun is not allowed to run his own clinic in Korea. While managing his own apothecary, however, he has actively expressed his opinions through printed media and an internet blog. In his second book about hwa-byung, Yun explained the illness by heavily relying on research about stress, strongly suggesting a holistic approach, and introducing teachings of Zen Buddhism. If the roots of hwa-byung and stress are associated with unfulfilled human desires, the ultimate treatment should come from a well-governed mind control. Interestingly, Yun often criticizes mainstream Korean traditional medicine’s narrow understanding of major Chinese classics.

Finally, but not least importantly, Kang Yong-wŏn introduces culturally embedded counseling as a major therapy for hwa-byung. Kang agrees with Kim in viewing the illness as a Korean manifestation of depression. Criticizing DSM-IV for its superficial approach to hwa-byung, Kang highlights that the illness represents a collective trauma that is deeply associated with Korea’s unprecedented struggle into modernity. Employing terms like *han *and *chŏng*, Kang illustrates that Koreans have not had enough opportunity to treat the emotional problems caused by fast economic development, a competitive educational system, and political vicissitudes (Kang 2011, p. 252). Furthermore, the suppression of emotion signifies the fundamental problem of Korean linguistic convention—the radical division between elite language (Chinese in the past and English in the present) and vernacular. It is Kang’s view that the elites in Korean history (at least since seventeenth century) governed their people by monopolizing the written language, degrading any verbal expressions that could not be transformed into classical Chinese. Although the Korean alphabet became the national language in the early twentieth century in line with the growing nationalism and colonialism, yet the gap between written and spoken languages deterred Korean medical experts from fully grasping the expressiveness of the bodily complaints by lay people. Kang argues for the possibility of turning to Korean vernacular with an in-depth knowledge of its semantics and hermeneutics to recover the locally inspired technique of therapeutic colloquiality.

Authors of recent popular volumes about hwa-byung share a common ground in utilizing the illness label to foster their own clinical intervention. Almost no one denies that hwa-byung is a uniquely Korean phenomenon (Hwang 2011, p. 11; Ch’oe et al. 2009, p. 5). As hwa-byung is fundamentally coined with Korean locality, Kang argues that doctors should diagnose not merely an individual patient but rather the entire “inventory” of Korea culture (Kang 2011, p. 254). All of these views overlap with Min’s earlier intention to find out the cultural root of hwa-byung in Korean patients.

Given the emphasis on indigenousness, however, Hwang, Ch’oe, and Yun do not specify how the unique elements of Korean culture are intertwined with the cause, manifestation, and treatment of the illness. Rather, they tacitly equate hwa-byung with stress, thereby establishing a means to utilize theories around stress in rationalizing hwa-byung for lay audiences. For instance, Hwang portrays hwa-byung as a state of excessive emotion, particularly caused by anger and anxiety. The overemotional state may cause a range of stress-related diseases. Although intended as a discussion of hwa-byung, more than a half of his book depicts skillful management of stress in everyday life. Each chapter fleshes out how stress causes circulatory, respiratory, digestive, endocrine organ, musculoskeletal, skin, genito-urinary, and children’s diseases. Based on this organization, Hwang’s explanation competently utilizes a range of biomedical terminologies. Except for the brief introduction to his preferred prescription, (p. 64) and his short comment on “Constitutional medicine” (appendix 1), no theories of traditional medicine are introduced.

Ch’oe and Yun show a similar trend with Hwang. Ch’oe puts both hwa-byung and stress in his title and regards them as the root of hundreds of diseases. The organization of Ch’oe’s book does not distinguish the symptoms and treatment of hwa-byung from those of stress. By taking “stress” as a medium, Choi relates hwa-byung with other diseases such as high-blood pressure, obesity, stroke, learning disability, sexual impotence, irritable colon syndrome, and other chronic illnesses. Yun also interprets hwa-byung as accumulated stress. Due to Yun’s personal inclination to Zen Buddhism, his text provides more general advice to control human desires and to cultivate one’s mind. Unlike the other three authors, Kang views hwa-byung as a Korean manifestation of depression. Yet, Kang overlaps with other authors in competently employing biomedical terms for his explanation.

In summation, recent popular texts about hwa-byung are consonant with the earlier studies of Min and Kim. All of the four exemplary authors agree regarding the indigenous nature of hwa-byung and compose the symptoms and treatments of the illness by referring to the DSM-IV definition, stress studies, and depression research. The link with stress is not entirely new. Kim, in his earlier studies, intended to benefit from stress studies by emulating the measurement scale (Lim et al. 2000). Kim’s desire to “scientize” hwa-byung is also resonant with the authors’ strategic management of bio-medical terms and weak elaboration of traditional medicine. Interestingly, the “Hwa-byung diagnostic interview schedule (HBDIS),” which was newly designed by Kim, has not been well received by the examined authors. In addition, Kim’s argument to view hwa-byung as Korean manifestation of depression found only one advocate, Kang.

Given this adjusted composition of hwa-byung by doctors of traditional medicine, more thought should be given to the role the illness label plays in criticizing the contemporary landscape of Korean medicine. If stress and depression aptly replace the manifestation of hwa-byung, why is the label still being used not only among patients but also among health specialists? What kinds of desires and complaints are associated with the contemporary use of hwa-byung as a meaningful disease name?

One of the most explicit demands by the above authors is to include patients’ life narratives into a treatment process and establish a better relationship between doctor and patient. Treating hwa-byung primarily implies fully delving into patients’ life stories, and accordingly, all the authors take patients’ first-hand narratives as a meaningful starting point for treatment. For instance, Hwang begins his book by narrating more than 31 cases that appealed for or were diagnosed as hwa-byung (Hwang 2011, pp. 14–56). Although they can be viewed as general problems of family conflicts, career failures, death of a beloved, and economic loss, every case has its own unique manifestation. To understand hwa-byung more fully, it is indispensable for doctors to listen to the stories carefully and to respond to them accordingly. This technique of listening is key. According to Hwang, today’s clinical environment is much too specialized and standardized, alienating both patients and doctors (Hwang 2011, appendix 4). He states, “The hesitant patient who does not know which department s/he should go for her/his health concern exemplifies one case of human alienation. Today’s clinics forget the wholeness of human beings and tend to serve as a repair shop. Besides, the more medicine develops, the more people are alienated. The clinic as a part feeder becomes corporatized and automatized (Hwang 2011, appendix 4).” Hwang hoped to recover a better patient-healer relationship. As a solution for hwa-byung, Hwang envisions better clinical communication that goes beyond simple questionnaires, tables, and numbers.

Yun agrees with Hwang in emphasizing the significance of dialogues between a healer and a patient. As Yun is not a licensed doctor of traditional medicine, the range of conversation Yun suggests includes more general principles of self-control, psychotherapy, and religious teachings. Interestingly, Yun allots almost one-third of his book to introducing the “91 Stories of Zen (禪話91則)” as a resource for soothing hwa-byung (Yun 2007, pp. 171–239). Although Yun does not specify how the Zen stories had impact on treating the illness, he argues that the teaching of Zen is a reliable means to control stress and hwa-byung. Yun believes the stories of illness can only be cured by another set of narratives; hence, the resonating healing power of Zen messages needs to be introduced.

Kang prioritizes narratives in elaborating his theory about hwa-byung. He argues: “To sum up, our life is nothing but stories, and depression is also a story. It is clear that treatment should be a story (Kang 2011, p. 175).” His narrative-centered approach calls for counseling that is based on a refined understanding of Korean vernacular. Kang attempts to analyze the linguistic convention of ordinary Koreans, not that of elites, to develop “medicine carried out by our own language, healing of counseling by our own language (Kang 2011, p. 189).” According to Kang, colloquial Korean puts emphasis on verbs, not nouns, which indicates the language’s full capacity to express human dynamics. Adjectives are rich; hence, the concrete details of individuality in things and people are aptly expressed. Furthermore, the use of the (non)substantive, a word order that highlights objects, the weakness of making logically connected long sentences, the development of immediacy and sensitivity, the lack of articles and prepositions, the significance of continuation, the qualitative approach, euphemism, and playfulness characterizes the unique nature of colloquial Korean (Kang 2011, pp. 189–199). Surely, not all scholars would agree with Kang’s characterization of Korean. What should be noted here is Kang’s problematization of the disjointed relationship between language and experience, which reflects the failure of the Korean medicine to fully come to grips with the expression of patients’ suffering. To reach the root of hwa-byung/depression, doctors, as healers, need to develop well-trained listening skills with an empathetic mind. Kang insists that “inter-subjectivity” between doctor and patient will help establish a more corresponding and meaningful relationship in clinical encounters.

Kang’s call for vernacularization of Korean clinical language is resonant with Min’s claim for a Koreanized psychiatry in the late 1980s. Twenty-five years after Min’s first call for indigenization, and even after Min’s decision not to emphasize any ethnic attribute of hwa-byung, opting for a much universal term, “anger disorder,” Kang regurgitates the turn toward indigenousness, thereby conjuring up the alternative in treating Koreans’ emotional disorders. Both Min and Kang hope to understand the fuller and deeper meaning of hwa-byung, which requires an articulation of “Koreanness” in medicine. To Kang, this endeavor implies the recovery of clinical colloquiality in Korean vernacular. To a certain degree, the underlying desires to make hwa-byung visible parallels a rising demand to make patients’ stories known and establish them as a clinically meaningful repertoire for treatment. The unique, individual, and irreducible elements of patients’ experiences need to be fully acknowledged and analyzed with respect.

A recent report by *Los Angeles Times* shows another hunger for making untold stories told. Dr. Cho Man-Chul, who has run a mental health program for Asians and Pacific Islanders in Los Angeles for more than 20 years, found out that Korean immigrants who called themselves as hwa-byung patients held on to their experiences of the Los Angeles Riot on April 29 1992. Showing a range of symptoms, such as paranoia, delusion, depression, and anger, Korean immigrants have accumulated their unexplained resentment and anxiety for dozens of years. Instead of moving on, they turned their anger inward, made it a part of their narrative for mundane, and continued calling it hwa-byung (Banks 2012). Cho put forth the significance of counseling, the process of speaking out and listening to, attempting to make the unaccountable accountable. Although Cho trained in Western psychiatry, he gradually became a representative of hwa-byung treatment. Cho insists that treating hwa-byung is nothing but establishing a long-term and reliable relationship of talking and listening. As a listener, Cho not only has felt a responsibility to record patients’ stories, but also hopes to make those stories available for later generations. That is why Cho first contacted the reporter to listen to the patient stories he has kept for 20 years.

## Epilogue

Contemporary medical discourses on hwa-byung reveal neither the success of biomedicine nor the essence of Korean culture. Rather, the project of defining a uniquely Korean malady reflects a desire among medical professionals to make the indigenous meaningful, thereby guaranteeing a tool for gaining circulation and foreign recognition. A closer look at the studies of Korean doctors of traditional medicine and psychiatrists reveals how temporary and unstable the ethnic label was in their portrayal of hwa-byung. The idea of the “uniquely Korean” emotional illness was first conjured up as a response to the rise of cultural psychiatry. Doctors of traditional medicine also stressed the Korean nature of the illness, arguing that their profession’s familiarity with Korean culture made them better qualified for effective treatment. However, after years of research, Korean medical professionals are disinclined to argue for the ethnic attributes of hwa-byung. The Korean distinction as an analytical unit was favorably employed at first, only tentatively used, and then erased from the discourse. The idea of the uniquely Korean attributes of hwa-byung has never been established by a clear consensus. Despite this fragility, however, the label hwa-byung continues to be utilized by medical professionals as the term partly helps them to account for patients’ life stories more fully and immediately. Embracing narratives as a therapeutic resource, a group of hwa-byung specialists is criticizing mainstream medicine’s lack of colloquiality and suggesting better techniques of patient-doctor dialogue. Interestingly, the label of hwa-byung signifies a turn toward the indigenous initiative, and now opens room for Korean doctors to engage with more general concerns of medicine, such as to respect the stories of illness, and to become better physician listeners.

## Footnotes

1 Constrained fire is Romanized as *ulhwa* (Korean) or *yuhuo* (Chinese). I’ll use Chinese Romanization “*yu*” for constraint hereafter to avoid any confusion with the term when it appears in other articles in this volume. For fire-illness, I’ll use “hwa-byung,” the Korean Romanization, instead of the Chinese “*huobing*.”

2 For more details about hwa-byung in Korean court records, see Kim 1999.

3 For a bibliography and a brief introduction to past research, see Min 2009a, b or Min 2011, especially pp. 3–4.

4 Yi is a well-known psychiatrist, popular author, and now a director of a healing center in Korea. He was affiliated with one of the two most privileged private universities in Korea and has played a role in popularizing hwa-byung since the 1970s.

5 Min Sung-kil is one of the most well-known specialists of Hwa-byung. Since the 1980s, Min has published dozens of articles and a book about Hwa-byung, calling for Korean psychiatrists’ attention to this seemingly indigenous illness.

6 Min addressed that his modification was largely centered on translating the questionnaires into familiar terms for Koreans or vice versa. An interview with Min, Aug 7 2012.

7 For instance, see Kim et al. (2007, pp. 60–61). Later research about hwa-byung published in *Journal of Oriental Neuropsychiatry*, *Journal of Korean Medical Association*, and *Journal of Korean Neuropsychiatric Association* often relies on Min for the definition of hwa-byung.

8 Traditional medicine in Korea seized the initiative by appealing to post-colonial nationalist agendas. As Korea was liberated from Japan in 1945 and announced the establishment of the government of the Republic of Korea in the South in 1948, how to structure medical professions became central to institutionalizing the health care system. During the 1950s, members of the National Assembly who trained in biomedicine refused to acknowledge the efficacy of traditional medicine, condemning its lack of scientific foundation as a medical theory. Against this opinion, advocates of traditional medicine emphasized that nothing is more suited to the bodies of Koreans than traditional medicine. Advocates also claimed that traditional medicine would ultimately contribute to the national health care system by complementing biomedicine. Their claim gained support; hence, the National Medical Services Law (Kungmin ŭiryobŏp) approved two medical systems in Korea on September 25, 1951; this plural system survives to the present day. Based on this law, medical careers were classified in three categories, as a biomedical doctor and dentist, as a doctor of traditional medicine, or as a health care worker, nurse, or midwife (Song 1995, p.28). Accredation of educational institutions was enhanced during the 1960s. The College of Oriental Medicine (Tongyang ŭiyak taehak), which was the only institution available for a career in traditional medicine at that time, finally qualified and was raised to the status of a university in 1964 with a 6-year educational program. Reflecting the increased interest in traditional medicine, more educational institutions have been established in the major cities since the 1970s. Today, 11 colleges of traditional medicine produce more than 700 graduates per year nationwide, and the total number of licensed doctors of traditional medicine in Korea amounted to 10,810 in 2008. The status of traditional medicine in Korea has improved legally and academically since the 1960s, and most agents of traditional medicine have not felt threatened by the authority of science. Rather, they have enhanced the scientific foundation of traditional medicine by including biomedical subjects, such as anatomy, physiology, biochemistry, and genetics, in the undergraduate curriculum (Suh 2007, p. 11).

9 Regarding the specialization of traditional medicine in Korea, see related topics published by the *Minjok Medicine News* (*Minjok* *ŭihak sinmun*, www.mjmedi.com, last visited on Sep 9, 2012). For instance, Chang-ho Han’s column on February 16, 2012.

10
www.ksdb.or.kr last visited on Sept 1, 2012.

11 Kim has played a leading role in establishing hwa-byung studies in Korea for last 20 years. See, for instance, www.hwabyung.kr (last visited on Dec 23, 2011).

12 Camile Lloyd, “Life events and depressive disorder reviewed: events as predisposing factors,” *Archives of General Psychiatry* 37:5 (1980), pp. 529–535, Thomas H.Holmes and Richard H. Rahe, “The Social Readjustment Rating Scale,” *Journal of Psychosomatic Research* 11:2 (1967), pp. 213–218, see Lim (2000), pp. 53–54.

13 See, for instance, www.omstandard.com (last visit on Dec 11, 2011).

14 Traditional medicine’s effort to establish an objective and standardized guide for diagnosis continues after the completion of HBDIS. See Kim et al. (2009, 2010) and Cheng et al. (2010).

15 See, for instance, Kim and Lee (2004), Ryu (2004), Lee et al. (2007), Chang (2009).

16 See Kim and Whang (1994) and Kim and Whang (1998).
